# Evaluating causal relationships between urban built environment characteristics and obesity: a methodological review of observational studies

**DOI:** 10.1186/s12966-014-0142-8

**Published:** 2014-11-18

**Authors:** Adam Martin, David Ogilvie, Marc Suhrcke

**Affiliations:** Health Economics Group and UKCRC Centre for Diet and Activity Research (CEDAR), Norwich Medical School, University of East Anglia, Norwich, UK; MRC Epidemiology Unit and UKCRC Centre for Diet and Activity Research (CEDAR), University of Cambridge, Cambridge, UK; Centre for Health Economics, University of York, York, UK

**Keywords:** Built environment, Natural experimental studies, Land use, Obesity, Evidence synthesis, Causal inference, Neighbourhood self-selection, Physical activity

## Abstract

**Background:**

Existing reviews identify numerous studies of the relationship between urban built environment characteristics and obesity. These reviews do not generally distinguish between cross-sectional observational studies using single equation analytical techniques and other studies that may support more robust causal inferences. More advanced analytical techniques, including the use of instrumental variables and regression discontinuity designs, can help mitigate biases that arise from differences in observable and unobservable characteristics between intervention and control groups, and may represent a realistic alternative to scarcely-used randomised experiments. This review sought first to identify, and second to compare the results of analyses from, studies using more advanced analytical techniques or study designs.

**Methods:**

In March 2013, studies of the relationship between urban built environment characteristics and obesity were identified that incorporated (i) more advanced analytical techniques specified in recent UK Medical Research Council guidance on evaluating natural experiments, or (ii) other relevant methodological approaches including randomised experiments, structural equation modelling or fixed effects panel data analysis.

**Results:**

Two randomised experimental studies and twelve observational studies were identified. Within-study comparisons of results, where authors had undertaken at least two analyses using different techniques, indicated that effect sizes were often critically affected by the method employed, and did not support the commonly held view that cross-sectional, single equation analyses systematically overestimate the strength of association.

**Conclusions:**

Overall, the use of more advanced methods of analysis does not appear necessarily to undermine the observed strength of association between urban built environment characteristics and obesity when compared to more commonly-used cross-sectional, single equation analyses. Given observed differences in the results of studies using different techniques, further consideration should be given to how evidence gathered from studies using different analytical approaches is appraised, compared and aggregated in evidence synthesis.

## Introduction

The global prevalence of obesity has increased in recent decades [1,2]. A contributing factor could be changes to the urban built environment, including suburbanisation (urban sprawl), which have altered the availability of a variety of dietary and physical activity resources. The costs (including time costs) of walking and cycling are likely to be higher in cul-de-sac housing developments, for example, compared to densely populated urban areas with greater land-use mix and shorter distances between home, leisure, retail and work locations. Fewer footpaths (sidewalks) and cycle routes would likely reinforce this cost differential. However, a potential counterbalance to high physical activity costs in suburban areas may be relatively low costs of accessing healthy foods, which are more readily available in larger out-of-town supermarkets (stores), at least in the U.S. [3]. Fewer public transport facilities and less road traffic congestion may also affect the costs of physical activity, although their impact could operate in either direction in different contexts. Policymakers seeking to reduce the (relative) costs people face when choosing healthy behaviours might therefore choose to intervene in the design of urban built environments.

Existing reviews — such as the review by Feng and colleagues [4], hereafter the ‘Feng review’ — document a substantial number of cross-sectional observational studies of the relationship between urban built environment characteristics and obesity using single equation regression adjustment techniques. Typically these reviews do not distinguish between these more common study designs [5,6], which can be used to test statistical associations and generate causal or interventional hypotheses [7,8], and other studies that may (at least in principle) strengthen the basis for causal inferences and provide a better guide for policymaking.

In particular, more advanced analytical techniques have been proposed in recent UK Medical Research Council guidance [9] (hereafter “MRC guidance”; Table 1) on evaluating population health interventions using natural experiments, in which variation in exposure to interventions is not determined by researchers. These include difference-in-differences (DiD) [10,11], instrumental variables [12,13], and propensity scores [13–15], which are intended to mitigate bias resulting from differences in observable or unobservable characteristics between intervention and control groups. Such methods have been used extensively by economists in observational studies to evaluate public policies that are typically not tested in randomised experiments [16].

**Table 1:** **Analytical techniques included in Medical Research Council guidance on natural experimental studies** ^**1**^

**Analytical technique**	**Brief description**
**Controlling for observable characteristics**	
Matching	Involves finding unexposed individuals (or clusters of individuals) which are similar to those receiving the intervention, and comparing outcomes in the two groups
Regression adjustment^2^	Measured characteristics that differ between those receiving the intervention and others can be taken into account in multiple regression analyses
Propensity scores	An estimate of the likelihood of being exposed given a set of covariates, propensity scores are usually estimated by logistic regression, and can be used to match exposed with unexposed units (which may be individuals or clusters of some kind) using values of the propensity score rather than the covariates themselves
**Controlling for unobservable characteristics**	
Difference in differences	Involves comparison of change over time in exposed and unexposed groups, which enables control of unobserved individual differences and common trends
Instrumental variables	An instrumental variable is a factor associated with exposure to an intervention, but independent of other factors associated with exposure, and associated with outcomes only via its association with exposure
Regression discontinuity	This approach exploits a step change or ‘cutoff’ in a continuous variable used to assign treatment, or otherwise determine exposure to an intervention. The assumption is that units (individuals, areas, etc.) just below and just above this threshold will otherwise be similar in terms of characteristics that may influence outcomes

^1^Source: Medical Research Council [9].

^2^For the purposes of the review, cross sectional studies that used single equation regression adjustment were excluded since they feature extensively in existing reviews.

These techniques can reduce the risk of ‘allocation bias’ (also known as ‘residual confounding’ in epidemiology [17] and ‘endogeneity’ or ‘self-selection bias’ in economics) which may arise particularly in observational studies [18,19] if people’s decisions about where they live are correlated with unmeasured individual-level characteristics (e.g. attitude towards physical activity) and with the outcome(s) of interest (e.g. obesity) [6]. Whilst randomised experiments are considered the ‘gold standard’ study design for estimating the effect of an intervention, since observed effect sizes can generally be attributed to the intervention rather than to unobserved differences between individuals, they are infrequently employed in public health research [20–22]. Particular barriers to their use in built environment research include ethical and political objections to the random assignment of participants to neighbourhoods, or to the random assignment of neighbourhoods to receipt of interventions, alongside the difficulty of blinding participants to their group allocation and limiting the potential for participants to visit neighbouring areas. The more advanced techniques described in MRC guidance may therefore provide a more realistic, if hitherto under-used, alternative approach.

The objectives of the present study were (1) to identify studies of the relationship between urban built environment characteristics and obesity that have used more advanced analytical techniques or study designs, and (2) to explore whether the choice of methodological approach critically affects the results obtained. For instance, do more advanced analytical techniques consistently show a weaker association between the built environment and obesity than single equation techniques — as would be expected if, for example, people of normal weight are more likely to choose to live in more walkable neighbourhoods? Should this be the case, then researchers and policymakers need to consider how evidence gathered from studies using different analytical techniques is appraised, compared and aggregated in evidence synthesis.

## Methods

### Search strategy

While recognising acknowledged difficulties in designing search filters on the basis of built environment characteristics [23], study design labels or design features across disciplines [24], a purposive search strategy was devised to elicit studies that may support more robust causal inferences than cross-sectional, single equation approaches. In order to identify additional studies to those included in the Feng review, a strategy was devised for the Ovid Medline (1950 to 2011) database encompassing a broader range of built environment search terms (based on another review [25]) and including papers published after 2009. Grey literature searches began with Google Scholar (to March 2013). On identifying a number of relevant studies published by U.S. economists at the National Bureau of Economic Research (NBER), the search was subsequently extended to the online repository of the NBER Working Paper series (http://www.nber.org/papers) and, to ascertain whether similar studies had been published in Europe, the online repository for research papers published by the Centre for Health Economics, York, U.K. (http://www.york.ac.uk/che/publications/in-house/).

The search was completed in two stages. In Stage 1, the search was restricted to observational studies using the more advanced analytical techniques identified in MRC guidance [9] (Table 1, excluding cross-sectional studies using only single equation regression adjustment since these feature in existing reviews).

In Stage 2, study designs or methodological approaches were identified which may not necessarily require use of the particular advanced analytical techniques specified in MRC guidance but may, nonetheless, support more robust causal inference. Specifically, this encompassed: (1) randomised experiments, (2) structural equation models (SEMs) [26], a multivariate regression approach in which variables may influence one another reciprocally, either directly or through other variables as intermediaries, and (3) panel data studies that controlled for fixed effects. In fixed effects panel data studies — as in those using the DiD approach — only changes within individuals over time are analysed, so eliminating the risk of bias arising from time-invariant differences between individuals (including in potential confounding variables) [27–29]. Other cohort, longitudinal or repeated cross-sectional studies which could not account for unobserved differences between individuals were excluded.

### Analysis

Data were extracted from each of the identified studies relating to the methods, including characteristics of the study population, the dependent and independent variables, analytical technique(s) and study design(s) employed; and to the results, including parameter estimates for one or more methods of analysis, noting any mismatch between the results of analyses that used different approaches.

## Results

### Objective 1: Characteristics of included studies

Of eight studies identified in Stage 1 of the review, all used instrumental variables and of these, six were cross-sectional and two were repeat cross-sectional studies (Table 2). Zick and colleagues, for example, used individual-level cross-sectional data on 14,689 U.S. women, linked to a walkability measure incorporating characteristics relating to land-use diversity, population density and neighbourhood design. An instrumental variable was derived from those characteristics (e.g. church or school density) that were significantly associated with the walkability of the neighbourhood but, crucially, not with BMI. In five of the eight studies, proximity to major roads (which was not correlated with BMI) was similarly used as an exogenous source of variation in relevant independent variables (e.g. fast-food restaurant availability (4/8), which increases around major roads because such amenities attract non-resident travellers). No studies identified in Stage 1 used the matching, propensity score, DiD or regression discontinuity (RDD) analytical techniques.

**Table 2:** **Results - observational studies identified in Stage 1 that used more advanced analytical techniques specified in MRC guidance (n = 8)**

**Study details**		**Description of variables**					**Results (for two different methods of analysis, when reported)**					
		**Independent variables**			**Dependent variables**		***Main method of analysis:***			***Alternative method of analysis:***		
							**More advanced analytical technique**			**Single equation analytical technique**		
**First author, date, journal**	**Study population**	**Description**	**Time varying**	**Areal unit precision**	**Description**	**Source**	**Description of analytical technique**	**Data type (time periods)**	**Effect sizes (95% confidence interval)** ^**1**^	**Method**	**Effect sizes (95% confidence interval)** ^**1**^	
											**Results where no statistically significant differences are observed between main and alternative analyses**	**Results where a mismatch between results is observed** ^**2**^
**Cross sectional studies**												
Anderson, 2011, American Economic Journal [30]	U.S. adults (11 States)	Miles between home and fast-food restaurant	N/A	Telephone/ZIP codes	BMI	BRFSS	Instrumental variable derived from distance to the interstate highway	Cross sectional (1)	0.09 (−0.17, 0.17)	Not reported		
Chen, 2012, Health Economics [31]	U.S. adults (Indianapolis, Indiana)	Number of	N/A	Individual addresses	BMI	Obesity Needs Assessment survey	Instrumental variable derived from distance to arterial roads and non-residential zones	Cross sectional (1)		OLS	None	Under-estimates:
		(a.) restaurants,							(a.) 0.37* (confidence interval missing)			(a.) 0.06 (−0.03, 0.14)
		(b.) chain grocery stores, and							(b.) 0.90* (0.12, 1.682)			(b.) 0.14 (−0.21, 0.50)
		(c.) proportion of park land, within a 0.5 mile radius							(c.) 2.85* (0.03, 5.67)			(c.) 2.39 (−0.66, 5.45)
Dunn, 2010, American Journal of Agricultural Economics [32]	U.S. adults (all States)	Number of fast food restaurants (at county level; author collected)	N/A	County level	BMI	BRFSS, 2004-2006	Instrumental variable derived from number of interstate highway exits in the county	Cross sectional (1)	No statistically significant results were reported, except in two subgroup analyses:	OLS	No statistically significant results were reported, except in two subgroup analyses (see right).	Under-estimates were reported in two subgroup analyses:
									Female participants in medium density counties: 0.06* (0.01, 0.11)			Female participants in medium density counties: −0.01 (−0.02, 0.01)
									Non-white participants in medium density counties: 0.20* (0.02, 0.38)			Non-white participants in medium density counties: 0.01 (−0.02, 0.04)
Dunn, 2012, Economics and Human Biology [33]	U.S. adults (Brazos Valley, Texas)		N/A	Individual addresses	Obesity likelihood	A mail survey	Instrumental variable derived from distance to nearest highway	Cross sectional (1)	No statistically significant results were reported, except in two subgroup analyses:	Probit model	No statistically significant results were reported, except in two cases (see right).	Under-estimates in just two cases:
									e.g. Non-white participants:		Non-white participants:	Non-white participants:
		(a.) miles to nearest fast-food restaurant, and number of fast-food restaurants within a							(a.) -0.100* (−0.178, −0.022)			(a.) -0.088 (−0.188, 0.012)
		(b.) 1 mile and							(b.) 0.189* (0.030, 0.348)			(b.) 0.052 (−0.021, 0.125)
		(c.) 3 mile radius							(c.) 0.058 (0.005, 0.121)			(c.) 0.014 (−0.004, 0.032)
Fish, 2010, Am J Public Health [34]	U.S. adults (Los Angeles County)	Resident perception of neighbourhood safety (self-reported dichotomous variable where 1= extremely or somewhat dangerous and 0=fairly or completely safe)	N/A	Individual level survey data	BMI	Los Angeles Family and Neighbourhood Survey	Instrumental variable derived from measures related to social cohesion and experience of household crime	Cross sectional (1)	2.81* (0.11, 5.52)	OLS (using first wave 2001/2 data)	None	Under-estimate: -0.07 (−1.07, 0.93)
Zick, 2013, IJBNPA [35]	U.S. females (Salt Lake, Utah)	Neighbourhood walkability	N/A	Census block (typically 1,500 people)	BMI	Utah Population Database	Instrumental variable derived from neighbourhood characteristics e.g. churches and schools	Cross sectional (1)	−0.24*	OLS	None	Under-estimate: 0.00
**Longitudinal studies**												
Courtemanche, 2011, Journal of Urban Economics [36]	U.S. adults (all States)	Number of Walmart Supercenters per 100,000 residents (these stores provide low cost food and encourage sedentary lifestyles)	Yes	County level		BRFSS, 1996-2005	Instrumental variable derived from distance to Walmart head office (expansion over time of Walmart stores was shown to be correlated with distance from the head office)	Repeated cross sectional (10)		OLS	None	Under-estimates:
					(i.) BMI				(i.) 0.24* (0.06, 0.41)			(i.) 0.02 (−0.00, 0.05)
					(ii.) Obesity likelihood				(ii.) 0.023* (0.011, 0.035)			(ii.) 0.001 (−0.001, 0.003)
Zhao, 2010, Journal of Health Economics [3]	U.S. adults (all States)	Proportion of people living in densely populated areas with >9000 people per square mile	Yes (4; every 10 years)	MSA level (366 of these in U.S.)	(i.) BMI	National Health Interview Survey, 1976-2001	Instrumental variable derived from exogenous expansion over time of the U.S. interstate highway system	Repeated cross sectional (25)	(i.) −0.01 (−0.03, 0.01)	Not reported		
					(ii.) Obesity likelihood				(ii.) −0.0013* (−0.002, 0.000)^3^			

BMI: Body mass index measured in kg/m^2^ BRFSS: Behavioural Risk Factor Surveillance System dataset. MSA: Metropolitan Statistical Area.

OLS: Ordinary-Least-Squares.

^1^ * indicates statistical significance at the p < 0.05 level.

^2^ when compared to results in the main analysis: “Under-estimate” if statistically significant results in the main analysis were not statistically significant the cross-sectional, single equation analysis; “Over-estimate” if statistically insignificant results in the main analysis were statistically significant in the cross-sectional, single equation analysis.

^3^ The interpretation of this result is that for each additional percentage point decrease in the proportion of population living in the densely populated area, obesity is approximately 0.1–0.2 percentage points higher.

Of six studies identified in Stage 2 (Table 3), two were randomised experiments. In one, the ‘Moving to Opportunity’ (MTO) study [37], families living in public housing in high poverty areas of five U.S. cities were randomly assigned housing vouchers for private housing in lower-poverty neighbourhoods. Significant reductions in obesity likelihood were observed after five years amongst voucher recipients when compared to non-recipients. In the other study, the exposure (not administered by researchers) resulted from the random (and hence exogenous) allocation of first year students to different university campus accommodation [38]. Three further studies identified in Stage 2 were fixed effects panel data analyses. Sandy and colleagues, for example, studied the impact of built environment changes in close proximity to individual households (derived from aerial photographs) on changes in the BMI of individual children over eight years. The sixth study was described as a structural equation modelling (SEM) study. Using cross-sectional data, physical activity and obesity status were modelled using latent variables for the physical and social environments [39].

**Table 3:** **Results - observational studies identified in Stage 2 that used alternative study designs or methodological approaches to support causal inference (n = 6)**

**Study details**		**Description of variables**					**Results (for two different methods of analysis, when reported)**					
		**Independent variables**			**Dependent variables**		***Main method of analysis:***			***Alternative method of analysis:***		
							**Panel data, RCT or SEM**			**Cross-sectional analysis**		
**First author, date, journal**	**Study population**	**Description**	**Time varying**	**Areal unit**	**Description**	**Source**	**Description of study design**	**Data type (time periods)**	**Effect sizes (95% confidence interval)** ^**1**^	**Method**	**Effect sizes (95% confidence interval)** ^**1**^	
											**Results where no statistically significant differences are observed between main and alternative analyses**	**Results where a mismatch between results is observed** ^**2**^
Franzini, 2009, Am J Public Health [39]	U.S. children (all States; 10–12 year olds)	Traffic levels, physical disorder, residential density and land use	N/A	Individual Systemic Social Observations	BMI	Interviews with students and their parents, 2003	Structural equation modelling (SEM)	Cross sectional (1)	0.03 (−0.40, 0.46) (these results relate to physical activity z-scores which contributed to the SEM. Physical environment had no significant impact on physical activity or BMI in the model)	Not reported		
Gibson, 2011 [40], Am J Public Health	U.S. young people (all States)	Five measures relating to food environment, including:	No	Zip-code level	BMI (obesity likelihood was also reported)	NLSY, 1998-2004	Fixed effects panel data analysis	Longitudinal data (2)	Change in BMI:	OLS	None	Under-estimates:
		(a.) supermarkets per square mile							(a.) -1.98* (−1.94,-2.02)			(a.) -0.04 (−0.18, 0.10)
		(b.) small grocery stores, and per square mile							(b.) -0.15* (−0.33,0.04)			(b.) 0.02 (−0.00, 0.04)
		(c.) full-service restaurants per square mile							(c.) 0.20* (0.03, 0.36)			(c.) -0.00 (−0.01, 0.01)
Kapinos, 2011 [38], Journal of Adolescent Health	U.S. undergraduate students (a single university campus)	Characteristics of dormitory accommodation:	No	Specific to the location of the dormitory accommodation	Weight (kg) (other outcome relating to exercise frequency, meals and snacks are not reported here)	Individual-level survey instrument (39 questions)	Randomised experiment (undergraduates were randomised to different dormitory accommodation)	Cohort data (2) One-year follow-up	Male (M) and female (F) participants:	Not reported		
		(a.) on-site dining hall							(a.) M: 0.19 (−2.37, 2.76) F: 0.85* (0.12, 1.57)			
		(b.) distance to gym							(b.) M: -0.25 (−1.37, 0.87) F: 0.13 (−0.32, 0.59)			
		(c.) distance to central campus							(c.) M: -0.08 (−0.80, 0.63) F: -0.45 (−1.15, 0.25)			
Kling, 2004, National Bureau of Economic Research [37]	U.S. (five cities; families with children; 85% with African-American or Hispanic female as household head)	Moving from a high poverty (public housing area) to a low poverty (a census tract with a poverty rate of less than ten percent) neighbourhood	No	Poverty rate was measured at the census tract level	Obesity likelihood	Individual-level survey	Randomised experiment: (moving to low poverty areas)	Cohort data (2) Five-year follow-up	(a.) intent-to-treat effect i.e. effect of being offered a housing voucher or the average effect of an attempted policy intervention on the entire target population:	Not reported		
									−0.048* (−0.091, −0.005)			
									(b.) treatment-on-treated i.e. those who moved using voucher			
									−0.103* (−0.195, −0.011)			
Powell, 2009, Journal of Health Economics [41]	U.S. young people (all States)	Measures included:	No	County level	BMI	NLSY, 1997-2000	Fixed effects panel data analysis	Panel data (4)	No statistically significant results observed in any of the measures. e.g.:	OLS	No statistically significant results observed except in one case (see right). e.g.:	Over-estimate in one case:
		(a.) restaurants per 10,000 people,							(a.) -0.03 (−0.09, 0.02)		(a.) 0.03 (−0.03, 0.09)	
		(b.) grocery stores per 10,000 people							(b.) -0.03 (−0.11, 0.05)		(b.) -0.0074 (−0.10, 0.08)	
		(c.) physical activity facilities per 10,000 people							(c.) -0.12 (−0.2, 0.05)		(c.) -0.16* (−0.30,-0.02)	
Sandy, 2009, National Bureau of Economic Research [42]	U.S. young children (Indianapolis, Indiana)	Twenty different measures,^3^ including:	Yes	Individual addresses	BMI (z scores)	Clinical records, 1996-2006	Fixed effects panel data analysis	Panel data (10)	In general, very few statistically significant results^3^	Cross-sectional OLS	In general, very few statistically significant results.	Over-estimates in two cases^3^:
									However, some selected exceptions (within 0.25 miles and including children of all ages, unless otherwise stated):			
		(a.) restaurants							(a.) -0.08* [−0.13 at 0.1 miles]			(a.) 0.02 [0.08* at 0.1 mile]
		(b.) supermarkets							(b.) 0.05 (0.1 miles)			(b.) -0.19* (0.1 miles)
												Under-estimates in three cases^3^:
		(c.)fitness,							(c.) -2.26*			(c.) 0.25
		(d.) kickball, and							(d.) -0.08*			(d.) 0.04
		(e.) volleyball facilities							(e.) -0.90* (0.1 miles; children <8 years only)			(e.) 0.03 (0.1 miles; children <8 years only)
												All within 0.25 miles and including children of all ages, unless otherwise stated

NLSY: National Longitudinal Survey of Youth dataset.

BMI: Body mass index measured in kg/m^2^.

OLS: Ordinary-Least-Squares.

^1^ * indicates statistical significance at the p < 0.05 level.

^2^ When compared to results in the main analysis: “Under-estimate” if statistically significant results in the main analysis were not statistically significant the cross-sectional, single equation analysis; “Over-estimate” if statistically insignificant results in the main analysis were statistically significant in the cross-sectional, single equation analysis.

^3^ Although 80 results were reported in total, the results reported in this table were for those variables deemed by the authors of that study to be most relevant to policy makers. Results were reported for four different sized areas/buffer zones (ranging from 0.1 to 1 mile).

In the five observational studies that used data from multiple time periods (two in Stage 1 and three in Stage 2), although BMI data were collected in up to 25 different time periods, data on built environment characteristics were collected less frequently and in three cases were fixed at a single time point. This could reflect the relative difficulty in collecting historical built environment data [29,43] which limits within-individual analysis to people who move location, rather than including those exposed to changes in the built environment around them.

Across both stages of the review, six studies (6/14, 43%) reported statistically significant relationships between built environment characteristics and obesity in the main analysis. Of these, four were instrumental variable studies identified in Stage 1 (statistically significant results were also reported for one of two obesity measures in one further study). Apart from the MTO study (for which the BMI results appeared only in the grey literature), all studies identified in the review were published after the Feng review had been completed in 2008, and all used data on U.S. participants. Nine studies (9/14) were published in sources that included “economic” or “economics” in their title.

### Objective 2: Comparison of results using different methodological approaches

Within-study comparisons of results were possible in six of the eight instrumental variable studies identified in Stage 1 (Table 2). In two of these studies [32,33], the results were statistically insignificant in both the instrumental variable and comparable single equation regression adjustment analyses. In four studies [31,34–36], statistically significant results reported in the instrumental variable analysis, in the expected directions, were not replicated in comparable single equation analyses. This was also the case in subgroup analyses such as for females or non-white ethnic groups in the other two studies.

Similar differences were also observed in one of the three panel data studies identified in Stage 2 of the review (Table 3) [40], as well as in some subgroup analyses of the panel data study by Sandy and colleagues in which statistically significant negative relationships between BMI and the density of fitness, kickball and volleyball facilities were statistically insignificant in the cross-sectional analysis.

These results suggest that use of cross-sectional, single equation analysis would have led to a lower estimate of the impact of built environment characteristics on obesity, whereas some authors had a prior hypothesis that these methods would have led to an overestimate of effect size arising from allocation bias. In contrast to an expectation that people of normal weight would prefer living in walkable neighbourhoods, for example, Zick and colleagues concluded that some neighbourhood features were positively associated with walkability and hence healthy living, but negatively related to other competing factors that people consider when choosing where to live, such as school quality, traffic levels and housing costs [35]. Similarly, although fast-food restaurants were expected to locate in areas with high demand [44], Dunn and colleagues suggested that a possible explanation for the statistically insignificant results identified in their instrumental variables study could be that these profit-maximizing firms operated in areas with low (not high) levels of obesity [32]. This may be because of higher average levels of education and income and lower levels of crime in those areas [33].

In contrast to the more common cases in which single equation, cross-sectional studies had relatively underestimated the impact of the built environment, in a small number of subgroup analyses of two of the panel data studies identified in Stage 2, statistically significant cross-sectional parameter estimates were not replicated in the panel data analysis (although in these two studies, the majority of parameter estimates were statistically insignificant regardless of the method of analysis) [41,42].

A more unexpected result in the study by Sandy and colleagues was the statistically significant negative relationship identified between the number of fast-food restaurants and BMI in the panel data analysis, which contrasted with a statistically insignificant estimate in the cross-sectional analysis. The authors did not suggest that fast-food restaurants actually reduced BMI in children, but concluded that a recent moratorium on new outlets in the U.S. city of Los Angeles might be ineffective, perhaps because outlets are already so commonplace that children can access fast food regardless of whether a restaurant is present in their immediate neighbourhood [42].

All remaining studies produced results that were in line with expectations. Furthermore, no studies were identified in which the application of at least two methods led to contradictory results (e.g. one estimate showing a positive and the other showing a negative impact).

In two of the instrumental variable studies identified in Stage 1 (2/8) [3,30], and in the randomised experimental and SEM studies identified in Stage 2 (3/6), results were not reported for any comparable alternative analyses.

## Discussion

### Objective 1: Use of more advanced methods

Despite increasing use of randomised experiments in policy areas where they are not normally expected [22,45–47], just two randomised experiments were identified in the review [37,38]. While RCTs ought not be overlooked as an evaluation option [48,49], the problem of “empty” systematic reviews would arise if non-randomised observational studies were excluded from evidence synthesis processes [50]. Scarce resources might then be diverted towards small-scale individual-level interventions [51], simply because RCTs of such interventions are more common, at the expense of large-scale population-level interventions, regardless of their relative cost-effectiveness [52].

The twelve identified non-randomised studies that used more advanced methodological approaches were all published during the past five years and, given that the Feng review identified 63 studies, already represent a sizeable contribution to the existing literature on the relationship between urban built environment characteristics and obesity. This indicates that, in the absence of evidence from RCTs, observational studies that employ the more advanced analytical methods are feasible and increasingly employed. In addition to their greater potential to support causal inference when compared to cross-sectional, single equation analyses, these observational studies may sometimes also provide more credible results than randomised experiments [53–57]. For example, large-scale, individual-level, retrospective data sets (e.g. the U.S. National Longitudinal Surveys (NLSY) and Behavioral Risk Factor Surveillance System (BRFSS), used in five studies) can potentially eliminate threats to internal validity likely to arise in public health intervention studies in which, unlike in placebo-controlled clinical trials, participants cannot be blinded to their group allocation. This can affect researchers’ treatment of participants [57] as well as participants’ behaviour and attrition rates. Although the impact on results was unclear, one-quarter of New York MTO participants were lost during follow-up, for example [58]. Further, in terms of external validity, larger sample sizes (e.g. Courtemanche and Carden’s study included 1.64 million observations [36]), longer follow-up periods, a wider range of variables relating to individual-level characteristics and the possibility of linking individuals to spatially referenced exposure variables identified in other datasets can support robust analysis of large, population-level interventions or risk factors, as well as smaller population-subgroup analyses [9]. In one such study, for example, statistically significant effect sizes were observed only amongst ethnic minorities [33]. These analyses are typically unfeasible in randomised experiments due to unrepresentative samples, high attrition rates, high costs or limited sample sizes. In Kapinos and Yakusheva’s study, for example, 386 students living in car-free campus accommodation, which was unrepresentative of external neighbourhoods, were followed up for just one year. Given an apparent mismatch in the schedules of experimental researchers and policy-makers [59], retrospective datasets can also support more rapid analyses and avoid the need for lengthy ethical approval processes associated with RCTs [45]. Nevertheless, all the identified studies featured U.S. participants (compared to 83% of the studies identified in the Feng review), which might be indicative of a scarcity of suitable datasets elsewhere, particularly in low- or middle-income countries [8].

Despite the apparent increased use of more advanced methodological approaches, not all the techniques recommended by the MRC for use in natural experimental studies featured in the identified studies. The absence of any study using the RDD or DiD approaches may be explained partly by a lack of suitable data and their relative inapplicability to built environment research, since policy interventions — particularly those involving the clear eligibility cut-offs that are required in RDD — may be relatively scarce. Further, most of the identified studies were published in economics journals, whereas none of the studies identified in the Feng review came from such sources. This could indicate the relative infrequency with which these techniques are used amongst public health researchers or are familiar to peer reviewers who are not economists [60]. However, in the case of propensity scores and matching, where the data requirements are similar to those of single equation techniques, some of their relative advantages over methods that control only for observable characteristics are not always acknowledged in existing guidelines [9]. First, they overcome the problem of wrongly specified functional forms, a recognised issue in built environment research [61]. Second, assuming that they are correctly applied [15], these techniques limit the potential for non-comparable individuals being included in the treatment and control groups [14,62,63] (problems related to their inappropriate use are highlighted in the next section). This so-called lack of ‘common support’ could be problematic if, for example, the most walkable neighbourhoods were home to individuals with levels of observed characteristics (e.g. higher income and education levels) that do not feature at all amongst the population of the least walkable neighbourhoods [14].

The review also revealed use of ambiguous or confusing study design labels — a recognised issue [24,64], owing perhaps to the relative novelty of natural experimental approaches. For example, ‘natural experiments’ are sometimes defined in broad terms as studies ‘in which subsets of the population have different levels of exposure to a supposed causal factor’ [65,66], or more narrowly, where ‘random or ‘as if’ random assignment to treatment and control conditions constitutes the defining feature’ [9,67]. Of the two studies identified that used “natural experiment” in their titles, the study by Sandy and colleagues only constitutes a natural experiment using the former definition [42]; the other, by Kapinos and Yakusheva, is better defined using the latter [38]. Yet these are not intervention studies and may therefore lie outside the scope of the natural experimental studies described in MRC guidance, despite their having exploiting variation which was outside the researcher’s control.

Established definitions of other terms, including fixed effects [68], quasi-experiments [6,64], DiD and SEM, may also vary between disciplines. In the present review, Franzini and colleagues used SEM to describe an observational study that used latent variables for the physical environment based on various built environment indicators [39], while Zick and colleagues [35], in common with other examples [69,70], used the term more broadly to encompass other multiple-equation analytical techniques, including instrumental variables. Elsewhere, the term SEM is used to describe a more specific research area which is distinct from the so-called ‘policy evaluation’ (or ‘reduced form’), multiple-equation methods that are the primary focus of the present paper [71,72]. Rather than evaluating specific interventions or policy changes and striving to develop techniques that mimic the RCT study design, structural models can be cumulative, incorporating existing theories and past evidence to simulate an array of potential built environment changes [73–75] and may therefore offer one promising but hitherto unexplored area for developing a better understanding of causal mechanisms and pathways in this field.

### Objective 2: Comparing effect sizes arising from different analytical approaches and implications for future primary research and guidance for evidence synthesis

Significant differences are — with some exceptions [76] — generally observed between the results of observational studies and randomised experiments [77–81]. However, comparisons of the results of observational studies that used different analytical techniques are uncommon. One unique series of studies in which different analytical techniques were used to evaluate the U.S. National Supported Work Demonstration programme, a 1970s job guarantee scheme for disadvantaged workers, is particularly insightful because statistically significant differences in effect sizes were observed when regression-adjustment, propensity score matching [82,83] and DiD [84] methods were used in analyses of comparable data arising from the same RCT [16,85].

One main finding of our review, that statistically significant relationships between features of the built environment and obesity were less likely when weaker, cross-sectional, single equation analyses were used, was unexpected, given the hypotheses of some authors (see Results section). Although this finding was based on a small number of within-study comparisons of results, it corresponds with a similar review of studies by McCormack and colleagues of the relationship between the built environment and physical activity which concluded that observed associations likely exist independent of residential location choices, an important contributor to allocation bias (although these studies focused primarily on using survey questions to elicit information about neighbourhood preferences and satisfaction, an approach that is associated with other sources of bias) [6]. A second main finding of our review was that 43% of identified studies reported statistically significant results in the main analysis, and that all statistically significant results were in directions that would be expected (except in one subgroup analysis). Although the estimated effect sizes were often still modest, a number of authors emphasised the potential of neighbourhood-level built environment interventions to influence the weight of large numbers of people [35]. Together with the Feng review which identified statistically significant effects in 48 of 63 studies (76%), these two main findings suggest that current interest in altering the design of urban built environments, amongst research and policymaking communities alike, seems warranted. Nevertheless, as in the two reviews by Feng and McCormack, the great heterogeneity in the range of built environment characteristics investigated limits the inferences that can be made about the specific changes to the built environment that are most likely to be cost-effective.

The finding that the use of different methods can make a difference to results suggests that, used appropriately, these more advanced methods should be considered as more robust approaches for establishing effect estimates of potentially causal associations between built environment characteristics and health-related outcomes. It also supports the case for improved tools to distinguish between studies in policy areas, including public health, criminology, education, the labour market and international development, where observational study designs are the norm [24,86–90]. Existing evidence synthesis guidelines, including MOOSE [91] and GRADE [92] used in health research and the Maryland Scale of Scientific Methods [93] which was developed by criminologists and forms the basis of recent guidance for U.K. Government departments [81,94,95], are not typically sensitive to potentially important sources of bias, including allocation bias, which may arise [78,90,96,97]. Meanwhile, more established tools, such as those developed by the Centre for Reviews and Dissemination [98], the Cochrane Collaboration [99] and PRISMA [100], focus solely on biases likely to be present in randomised intervention studies, including allocation concealment and attrition bias [99].

Nevertheless, enhancing these guidelines so that they are more sensitive to differences between different observational study designs would be challenging. First, unlike the common distinction between RCT and non-RCT intervention research, it is not generally possible to state that any analytical technique is universally preferable to another in all observational settings [84]. Rather, a researcher’s choice of technique should be based on pragmatic and subjective judgements dependent on the data available and the study context. In many cases, none of the advanced analytical techniques would be suitable, and rarely would they be interchangeable. Second, each analytical technique has distinct features which must be borne in mind when interpreting results. For example, instrumental variable analyses rely on subjective, unverifiable judgments about the quality of the instrument [74,101–104], and are therefore liable to be used inappropriately [60]. Reviewers of instrumental variable analyses must also consider the population subsample that has been used in the analysis [105,106] and, in propensity score analyses, of the characteristics of participants for whom there is common support [15,107]. Sometimes this detail is overlooked or left unreported by study authors [15]. Hence reviewers or policymakers may conclude that the results of comparable cross-sectional, single equation studies provide a more reliable guide, despite the associated risk of allocation bias. Reporting guidelines designed for authors of studies of observational studies (e.g. STROBE [108,109]) could be better developed [77] to alleviate inadequacies in the reporting of results, but also to encourage authors to report the results of a comparable single equation or cross-sectional analysis. Third, other important sources of bias may be overlooked if an assessment of study quality were based solely on the chosen analytical technique. Evident in the present paper, for example, were the use of self-reported rather than objectively measured BMI outcomes [4] and perceived rather than objectively measured characteristics of the built environment [110], differences in the strength of temporal evidence in longitudinal studies (i.e. whether a change in environmental characteristics actually preceded a change in obesity), varying attempts to control for residential self-selection using self-reported attitudes [6], and a trade-off between the use of large pre-existing administrative boundaries (e.g. the study by Powell and colleagues of adolescent BMI [41]) and more sophisticated approaches based on georeferenced micro-data (e.g. the study by Chen and colleagues [31]) (Tables 2 and 3). While the latter can provide a detailed description of each individual’s immediate living environment, a possible bias would likely arise if individuals engaged in dietary or physical activity behaviours outside their immediate area [111].

## Conclusion

Use of more advanced methods of analysis does not appear necessarily to undermine the observed strength of association between urban built environment characteristics and obesity when compared to more commonly-used cross sectional, single equation analyses. Although differences in the results of analyses that used different techniques were observed, studies using these techniques cannot easily be ‘quality’-ranked against each other and further research is required to guide the refinement of methods for evidence synthesis in this area.
